# Anticipated barriers and facilitators of implementing a digital behavioral treatment for chronic pain: a qualitative interview study

**DOI:** 10.1186/s12913-026-14820-8

**Published:** 2026-05-29

**Authors:** Afra Selma Taygar, Sara Laureen Bartels, Hannah Christie, Angeliki Pelika, Samira Hesse, Ida Flink, Suzanne Petersson, Sophie I. Johnsson, Katja Boersma, Lance M. McCracken, Rikard K. Wicksell

**Affiliations:** 1 Department of Clinical Neuroscience, Karolinska Institutet, Stockholm, Sweden; 2 Department of Psychiatry and Neuropsychology, Mental Health and Neuroscience Research Institute, Maastricht University, Maastricht, the Netherlands; 3 School of Population Health, Royal College of Surgeons in Ireland, Dublin, Ireland; 4 Leiden University, Leiden, the Netherlands; 5 Tilburg University, Tilburg, the Netherlands; 6 Centre for Psychiatry Research, Department of Clinical Neuroscience, Karolinska Institutet, Stockholm, Sweden; 7 Department of Medicine and Optometry, Linnaeus University, Kalmar & Region Kalmar County, Kalmar, Sweden; 8 Department of Molecular Medicine and Surgery, Karolinska Institutet, Stockholm, Sweden; 9 Center for Health and Medical Psychology (CHAMP), School of Behavioural, Social and Legal Sciences, Örebro University, Örebro, Sweden; 10 Department of Clinical Psychology, Uppsala University, Uppsala, Sweden; 11 Centre for Biomedical Research in Epidemiology and Public Health (CIBERESP), Madrid, Spain; 12 Pain Clinic, Capio St. Göran Hospital, Stockholm, Sweden

**Keywords:** Implementation, Barriers, Facilitators, Stakeholders, Digital intervention, Chronic pain

## Abstract

**Background:**

In digital health innovations, sustainable implementation should be considered from the initiation of the development to ensure that the solution fits into the existing healthcare system. This study aims at identifying barriers and facilitators for the prospective implementation of a digital behavioral intervention for chronic pain in the Swedish healthcare system, as perceived by key stakeholders.

**Methods:**

Eight semi-structured individual interviews were conducted with stakeholders (*n* = 2 IT developers, *n* = 4 healthcare managers, *n* = 2 healthcare professionals). Qualitative data were analyzed using a framework analysis following the Consolidated Framework for Implementation Research (CFIR).

**Results:**

A total of 86 facilitators and 38 barriers to prospective implementation were identified across stakeholder interviews. Facilitators included perceived relevance of the digital intervention and organizational readiness. Identified barriers primarily related to insufficient training and supervision, unclear decision-making processes (e.g., specific actions, responsibilities of stakeholders), and the lack of systematic needs assessment to guide implementation planning.

**Conclusion:**

Overall, findings indicate a high level of openness toward digital interventions within the implementation context. While implementation context largely demonstrates readiness, successful integration will depend on proactively addressing identified barriers. Awareness of these factors enhances the likelihood of successful and sustainable implementation.

**Supplementary Information:**

The online version contains supplementary material available at 10.1186/s12913-026-14820-8.

## Background

Chronic pain is defined as pain persisting or recurring more than three months, and affects approximately a quarter of the adult population worldwide [37]. The complex nature of chronic pain extends beyond persistent physical discomfort, often impacting an individual’s overall well-being and quality of life, including the ability to perform everyday activities, work, and social interactions [5]. Behavioral interventions informed by theoretical frameworks such as fear-avoidance and psychological flexibility models focus on improving resilience to pain and distress [21, 39]. While these approaches have a strong empirical foundation [10], access to such evidence-based behavioral interventions remains limited for many people with chronic pain [9].

Digital behavioral interventions offer a promising solution to the limited access to traditional face-to-face treatment, and may expand the reach of effective care for chronic pain [11]. Despite their considerable potential, successful implementation of evidence-based digital behavioral interventions into routine healthcare remains a significant challenge, due to various factors at the individual, organizational, and system levels. Individual-level barriers include limited technical skills and low user engagement [20]. Organizational challenges may be seen in insufficient staff training or support and misalignment with organizational structures [32]. At the system level, issues such as lack of infrastructure and inadequate integration with existing health information systems may pose challenges [31].

Identifying potential challenges early in the development of a new digital intervention is critical for effectively addressing factors that may influence the implementation process [7]. *Implementation* refers to the process of putting to use or integrating evidence-based interventions into a specific setting. Engaging and consulting stakeholders is recommended for interventions that are known to be effective so that these can be applied in ways that are feasible and sustainable in real-world settings [33]. As such, stakeholders can provide valuable insights and identify anticipated facilitators and barriers for successful and sustainable implementation [16]. Specifically, stakeholders may include end-users like patients, as well as healthcare professionals, managers, and IT developers.

Stakeholder engagement is central to the ‘DAHLIA’ (Digital behaviourAl HeaLth for chronIc pAin) program, a multiphase project with the overarching objective to develop, evaluate, and successfully implement a digital behavioral intervention for people with chronic pain into the Swedish healthcare system. Patient perspectives on the design and content of the intervention were explored in a separate study involving focus groups [35]. Here, focus is on gaining insights into the perspectives of stakeholders to explore systemic factors that may impact implementation.

The present study aims at identifying barriers and facilitators for the implementation of a digital behavioral intervention for chronic pain. Specifically, the perspectives of stakeholders (IT developers, healthcare professionals and healthcare manager) in one of 21 Swedish regions were explored. Finally, implementation strategies are considered to successfully navigate identified barriers and utilize facilitators.

## Methods

### Setting

The present study is a part of the multi-phase DAHLIA project with the objective of developing, evaluating, and implementing a digital behavioral intervention for people with chronic pain [2]. The digital intervention is intended to be implemented through the national healthcare web platform in Sweden, 1177 (1177.se), a nationwide digital infrastructure that provides people in Sweden with access to healthcare information, digital services, and secure patient and healthcare provider communication across all regions. The present study reflects on perceived barriers and facilitators to implementation according to stakeholders, while the development and piloting of the intervention is described in separate papers [35, 2]. In this manuscript, three stakeholder groups are defined. *IT developers* are individuals responsible for the technical and practical design, development, and maintenance of the digital behavioral intervention in the online platform. *Healthcare professionals* are individuals providing the digital behavioral intervention to people with chronic pain. *Healthcare managers* are individuals responsible for managerial or strategic decision-making related to digital health products, services, and/or processes within the Swedish healthcare system in Region Kalmar, Sweden. The term ‘patient’ is used interchangeably with ‘person with chronic pain’, as participants were recruited within a healthcare setting. The study was approved by the Swedish Ethical Review Authority (approval number: Dnr 2021–02437).

### Study design

A qualitative study with semi-structured individual interviews was conducted with stakeholders, including IT developers, healthcare managers, and healthcare professionals from Region Kalmar, Sweden. Individual interviews focused on exploring perceived facilitators and barriers of the prospective implementation of a digital behavioral intervention for chronic pain in the Swedish healthcare system.

### The digital behavioral intervention for people with chronic pain

This study focused on prospective *implementation determinants*, i.e., underlying factors that influence whether an intervention can be successfully implemented, and a treatment vision for the digital behavioral intervention has been established [2]. The DAHLIA intervention consists of self-guided micro-sessions, i.e., brief, frequent, and structured digital intervention components, combined with weekly therapist contact (e.g., chat function, phone call, video call). The intervention is intended to be delivered through the Swedish healthcare system and integrated into 1177.se, the national healthcare web platform in Sweden.

### Participants and recruitment

Eight interviews were conducted, to include a variety of perspectives [2]. The sample size was guided by the concept of information power, where a smaller number of participants is sufficient when the study aim is specific and participants hold relevant expertise [19]. In this study, participants were purposefully selected to present different key stakeholder groups involved in the implementation, ensuring variation in perspectives across roles. Thus, perspectives (i.e., the researchers attempt to take into consideration difference perspectives of the research problem, which leads to rich insights [23] were used as an indicator for data saturation.

Interview participants were identified through convenience and snowball sampling (i.e., directly contacted by researchers or referred to by previous participants). A research assistant (SIJ) emailed participants the study information and a link to provide informed consent digitally via REDCap (Research Electronic Data Capture) [14, 15]. Following informed consent, the interview was conducted online and lasted approximately one hour.

Participants represented three stakeholder groups involved in the implementation of the digital health intervention. Participating healthcare professionals were licensed psychologists or psychotherapists trained in cognitive behavioral therapy. Healthcare managers and IT developers were directly or indirectly working with digital innovations (e.g., decision-making on an organizational level, technical support). Participants were all working in Region Kalmar, Sweden. No other inclusion or exclusion criteria apply. In total, eight stakeholders participated in the study, including IT developers (2), healthcare professionals (2), and healthcare managers (4).

### Materials and procedure

The interviews explored stakeholders’ experiences and perceptions in relation to the implementation of the digital behavioral intervention, with a particular focus on barriers, facilitators, and contextual factors influencing implementation. The interviews were conducted as a recorded video meeting (MS Teams) between December 2021 and July 2022. Interviews followed a semi-structured format (see Multimedia Appendix 1 for full interview guides). The interview guides were developed based on the study aim and were informed by previous research on digital health implementation [6]. Interviews consisted of a set of questions tailored to each interviewee, focusing on their specific knowledge, role, and experiences. Specifically, (i) developers were asked about the implementation of digital interventions into the 1177.se web platform, (ii) healthcare managers discussed their experience with and support for existing digital interventions within healthcare system, and (iii) healthcare professionals reflected on their experience and use of digital interventions in clinical practice.

At the beginning of each interview, an overview of the DAHLIA study and the objective of conducting interviews was presented to participants. As stated in the informed consent, each participant was reminded that the meeting would be recorded and only the research team would have access to the recorded and transcribed data. Additionally, participants were informed that their responses would remain anonymous. They were encouraged to speak openly as the purpose of the interview was to learn from their experience.

### Data analysis

As the thematic framework, the Consolidated Framework for Implementation Research (CFIR) [7, 8] was used to guide the analysis of the qualitative interview data. CFIR consists of 39 constructs across five main domains: (1) intervention, (2) outer setting, (3) inner setting, (4) individuals, and (5) implementation process. The definitions of the domain adapted to the context of this study are presented below (Table 1).

The interviews were conducted digitally and transcribed verbatim. Six interviews were conducted in Swedish by a research assistant (SIJ) and professionally translated into English. The translated transcripts were reviewed by the research team to ensure accuracy and consistency with the original Swedish transcripts. Two interviews were conducted in English by a postdoctoral researcher (SLB). The transcripts were subsequently analyzed by non-native English speakers (AST, AP, SH). Each transcript was coded by two researchers, with AST involved in all transcripts and either AP or SH coding alongside. Refinement of coding and interpretation were conducted collaboratively, with regular discussions between coders to resolve discrepancies. The coding framework was refined throughout the analysis process. The authors acknowledge that most members of the research team were involved in the development of the DAHLIA intervention [35], which is to be expected in a multi-phase project [2]. Nevertheless, the authors acknowledge that this prior involvement may have influenced their perspectives (AST, SLB) during data analysis. Through team meetings and written feedback, additional perspectives shaped the refinement and interpretation of the results. The expertise of HC is emphasized as she was not part of the development and offered unbiased input.

While the study protocol [2] specified a use of thematic analysis with an inductive approach, the analytical strategy was refined during the course of the study (deviation from the protocol). Instead, a qualitative framework analysis was applied [28, 34] to allow for both inductive identification of themes and deductive mapping onto the CFIR, with the following steps: (1) *familiarizing* with the material by listening to recordings and reading transcripts, (2) *identifying* the thematic framework by combining an inductive and deductive approach (i.e., themes mapped onto the framework or add additional themes, and creating themes within framework), (3) *indexing* by creating a coding frame linked to the theoretical framework to highlight key points, (4) *charting* by placing themes into rows and columns in summary, and (5) *mapping and interpretation* by examining patterns of similarities and differences across participants’ responses. This approach was considered more appropriate given the study’s focus on implementation and the use of an established theoretical framework, enabling a more structured analysis.

**Table 1:** The adapted definitions of the CFIR domains in the context of the study

CFIR Domain	Construct definition
Intervention	The characteristics of a digital behavioral intervention for chronic pain prospectively implemented into the Swedish healthcare system
Outer Setting	The broader economic, social, and political context in Sweden that impacts the implementation of the digital behavioral intervention for chronic pain
Inner Setting	The context in which the digital behavioral intervention for chronic pain is implemented, specifically the 1177.se healthcare web platform and healthcare processes in primary care in Region Kalmar
Individuals	The roles and characteristics of individuals, specifically developers, healthcare managers, and healthcare professionals, that can be part of the prospective implementation
Implementation Process	The approaches and steps taken to implement the digital behavioral intervention for chronic pain in Region Kalmar

To enhance the trustworthiness, several strategies were employed. Investigator triangulation was achieved through the involvement of multiple researchers in the coding process. Regular discussions were held to compare interpretations and reach consensus, supporting the credibility of the findings. An audit trail was maintained through documentation of coding and iterative development of the coding framework. Study materials, including semi-structured interview guide and interview transcripts, further contributed to the transparency of the research process. The use of CFIR framework further supported a systematic approach to analysis.

## Results

### Facilitators and barriers of implementing a digital behavioral intervention for chronic pain in Region Kalmar

**Fig. 1:**
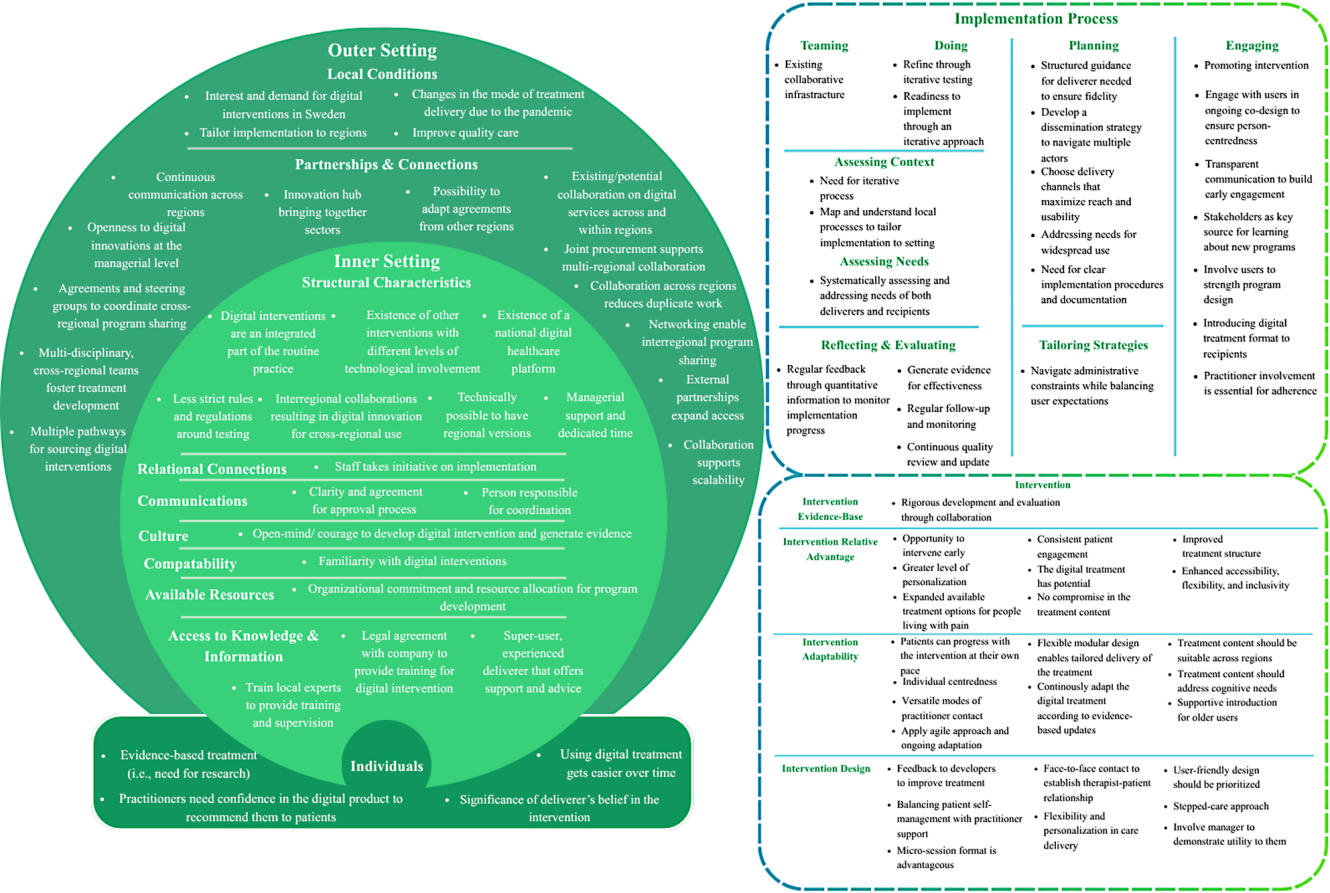
Facilitators identified in the interviews categorized according to the CFIR framework

**Fig. 2:**
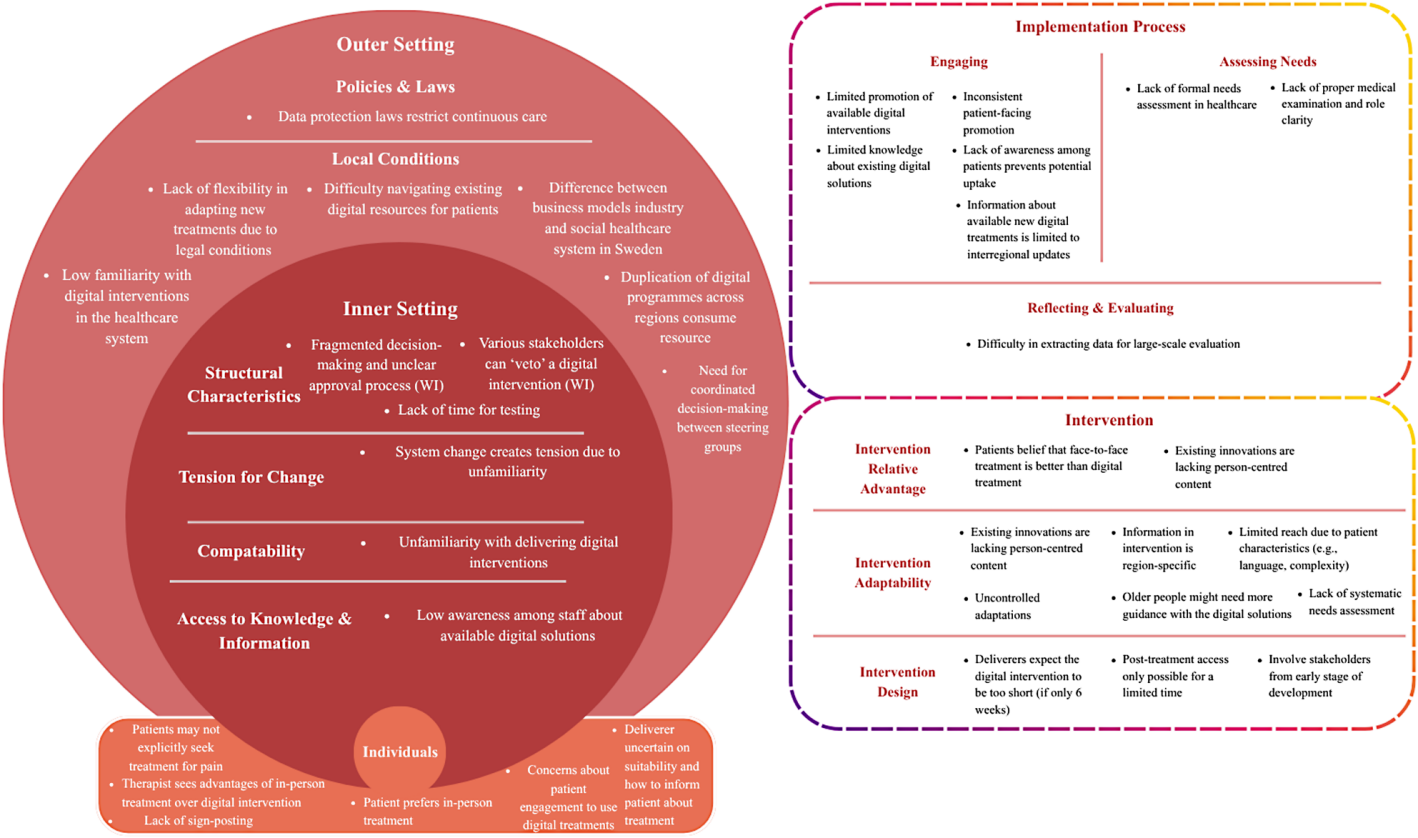
Barriers identified in the interviews categorized according to the CFIR framework

In total, *n* = 86 facilitators (Fig. 1) and *n* = 38 barriers (Fig. 2) were identified, connected to the intervention, outer setting, inner setting, individuals, and implementation process. Below, these factors are elaborated per CFIR domain. Themes are presented as italicized statements, reflecting key factors identified through the qualitative analysis.

### Intervention

#### Evidence base

The digital intervention for chronic pain planned to be implemented into the Swedish healthcare system was perceived by the stakeholders as *rigorously developed and evaluated through collaboration*, which was described as a factor that facilitated its implementation: *“[The digital behavioral intervention to be implemented] feels very solid*,* both in terms of experience and knowledge. (…) You are really trying to collaborate broadly so that it’s not just one person’s conviction of what’s best*,* but that you do it together simply.”*. Notably, participants did not express any barriers relating to the evidence base of the digital intervention.

#### Relative advantage

Compared to traditional care, several facilitators of the digital format of the intervention were emphasized. A key facilitator was seen in the *opportunity for a psychological intervention provided earlier* in the patients’ health care journey, as opposed to receiving the treatment after all other medical options were exhausted. The digital intervention was also considered to *expand available treatment options* for people living with pain and to offer a more *structured approach to treatment*, with all materials and exercises being available in one place. This facilitator was reflected in the interviews, with one stakeholder (Participant E) reporting that a digital version is *“convenient because you can sit anywhere and you can reach everyone*.“, highlighting its potential to broaden access to treatment. Moreover, they emphasized that digital format enables the *integrity of the treatment content without compromise*, allowing psychoeducation, psychological exercises, skills training and other therapeutic components to be delivered together.

Stakeholders noted as a barrier that *many existing digital interventions lacked person-centered content*, while highlighting that the present digital intervention was perceived as having a *person-centered structure* making it more flexible and better suited to varied needs of people living with pain, compared to other existing digital treatments. Another reported barrier was that some *patients might have a belief that face-to-face treatment is better than digital treatment*, which could impact their willingness to engage with the intervention: “*Sometimes people think that if they [have] face-to-face treatment*,* it will be a better treatment. Whereas I know*,* or can say*,* that in most cases it will not be a better treatment*” (Participant H).

Overall, the digital intervention was perceived as a facilitator of more *consistent patient engagement*, through regular check-ins and self-monitoring. Also, it was seen as *enhancing accessibility and inclusivity*, enabling more patients to receive care without the constraints of physical visits to the clinic. Thus, the intervention was considered by stakeholders to have *strong potential to improve treatment quality and outcomes*.

#### Adaptability

Stakeholders emphasized the importance of the ability to adapt to the diverse needs of people with chronic pain, and to the conditions in various healthcare contexts. One key facilitator of DAHLIA was the intervention’s *flexible*,* modular design that enables tailored delivery* to individual needs: *“It can be different paces*,* different amounts of content*,* different exercises*,* different amounts of contact with practitioners and so on. So*,* the flexibility is one of the key arguments for digital interventions in general*,* and DAHLIA”.* This flexibility included *versatile modes of contact* (e.g., chat function, phone call, video call), and how the treatment approach allows *patients to progress at their own pace*. Such features were considered essential for promoting *personalized* and *individual-centered care*, which implies that the *treatment can be suitable across different regions and healthcare settings*.

The significance of *applying agile*,* ongoing adaptations* as a facilitator for successful implementation was also mentioned. Stakeholders discussed the importance that the intervention should not be finalized in the sense of remaining static but instead be *continuously improved*,* adapting to new evidence*, in contrast to examples of existing digital interventions that have not been updated despite changes in the evidence. Additionally, stakeholders highlighted the need for the *treatment content to meet cognitive needs*, to ascertain it is easily understood by individuals with varying levels of cognitive abilities (e.g., attention and ability to focus while experiencing pain). In addition, providing *supportive introductions to the treatment* and additional guidance for patients, especially older individuals or those with low technical literacy, was considered essential.

Several barriers to adaptability were mentioned: The *lack of systematic prioritization and selection process based on identified patient needs* was noted as a potential barrier. Some stakeholders noted that new digital interventions usually emerge from individual enthusiasm (e.g., clinician, operations developer) or regional initiatives rather than from structured assessment of patient needs: *“You should perhaps start from the other end and see which patient groups would need digital support*,* and then start the development from there”* (Participant F). Stakeholders also highlighted that the intervention’s *reach could be limited by patient characteristics*, such as language barriers or complex chronic pain conditions. Specifically, the *limited language adaptability might restrict access to migrant groups*. Moreover, *older people with chronic pain might need more guidance* to engage with the digital intervention effectively. Stakeholders also pointed out that the existing digital interventions often *contain region-specific information*, which reduces the possibility of easy transferability across regions. Furthermore, there were concerns about the *risk of uncontrolled adaptations* (i.e., local stakeholders changing content or setup of the intervention) during national implementation, with stakeholders recommending that adaptations be guided to maintain the core components of the intervention.

#### Design

Stakeholders highlighted several aspects of the design of the intervention. The opportunity for providing *ongoing feedback from intervention deliverers on the design to developers* was considered as a facilitator to improve the intervention. Participants highlighted the importance of a *user-friendly design*, emphasizing the need for *accessibility*,* flexibility*, and *personalization* in the digital intervention. Another key facilitator was the *micro-session format* of the digital intervention, which was described as advantageous, allowing patients to digest the content easier and to integrate new behavioral patterns into daily routines:*“It’s not one part that you go through and then work with*,* for those who lose motivation during the week. I can see advantages of [delivering the intervention in micro-sessions] approach. And if it’s not so big each time*,* so that it feels okay to go in each time”*. In addition, a *stepped-care approach*, with an initial focus on unique individual needs, was emphasized to tailor the intervention while maintaining structure. Moreover, it was noted that an initial *face-to-face contact to establish the therapist-patient relationship* would be valuable to support patient trust. On a related note, stakeholders mentioned the significance of *balancing patient self-management with practitioner support* throughout the intervention. *Involving stakeholders from an early stage of development*, as well as *engaging managers to demonstrate utility* of the digital intervention, were considered essential.

Some stakeholders expressed concern that if *the digital intervention is offered as a short intervention (e.g.*,* six weeks)*, it might be insufficient for addressing the needs of the target population considering many people with chronic pain live with the condition for a long period of time. Another barrier was seen in the *limited post-treatment access*, as patients might only be able to revisit the content for a short period of time after completing the treatment; it was suggested that permanent access to the intervention could support help sustainable effects.

### Outer setting

The outer setting refers to the broader healthcare environment, including policies, inter-organizational relationships, and sociopolitical conditions that may influence implementation.

Stakeholders mentioned a strong *general interest and demand for digital interventions in Sweden*, further increased by the *changes in the mode of treatment due to the COVID-19 pandemic*. An *openness to digital interventions at the managerial and system level* was also emphasized. One facilitator was the *existing and potential collaboration on digital interventions across and within regions*, which also *reduces duplicate work* and allows for *continuous communication across regions to support implementation: “You don’t make a treatment program in 15 minutes*,* it can take six months or a year before it’s completed and tested and implemented. So now we see that we need to be better at collaborating with other regions to avoid duplicate work and to promote collaboration*,* where we do things together.”* (Participant G). Stakeholders highlighted that *joint procurement supports multi-regional collaboration* and that *multi-disciplinary*,* cross-regional innovation teams* foster successful intervention development. The *possibility to utilize agreements from other regions as templates*, and to establish *formal agreements and steering groups to coordinate cross-regional program sharing* was considered an implementation facilitator. Additionally, the existence of an *innovation hub bringing together healthcare*,* academia*,* and industry* facilitated collaborative development. Utilizing efficient *internal communication and external partnerships expands access*, *supports scalability*, and enables the *tailoring of implementation to specific regions*, allowing solutions to be adapted to local contexts.

Several barriers were mentioned by stakeholders. *Differences between business models within industry and those in social healthcare systems* may create ambiguity and uncertainty regarding e.g., revenue streams and reimbursement for digital health services: *“Companies’ approach does not always fit with health and social care’s agenda”*. Also, people involved in digital health innovation may work in different sectors, e.g., health care or industry, with different understanding of the context in which the digital health solution will be implemented.

*Duplication of digital programs across regions consumes resources.* Moreover, *lack of flexibility in adapting new treatments due to legal conditions* was thought to negatively affect the innovation process by increasing the time from prototype development to use in practice. Stakeholders also emphasized that *low familiarity with digital interventions in the healthcare system* might impact the adoption of the digital intervention by the deliverers: “*I think the Swedish healthcare system in general and also Region Kalmar are so unfamiliar with to [digital interventions]*” (Participant D). The *difficulty navigating existing digital resources for patients* was mentioned as a risk for reduced engagement.

### Inner setting

The inner setting captures organizational characteristics that may impact implementation, including infrastructure, internal processes, and available resources.

Stakeholders highlighted several factors that facilitate implementation within their organizations. A major strength was the *existence of a national digital healthcare platform* (1177.se), providing an infrastructure for the delivery of the digital interventions. The *existence of other interventions with different levels of technological involvement* creates *familiarity with the digital interventions*. It was noted that it is *technically possible to create different regional versions*, supporting local adaptation. *Organizational commitment and resource allocation for program development*, alongside a *person responsible for coordination*, were considered essential. Moreover, it was highlighted as a facilitator that *digital interventions are an integrated and well-accepted part of the routine practice* in the Swedish healthcare system. It was also recommended to identify patients at risk of dropouts or those who had already discontinued treatment. Stakeholders noted that they already *actively track compliance* and reach out to patients who stop logging in to a digital intervention to understand the underlying reasons.

It was furthermore emphasized that *interregional collaborations result in digital interventions that can be used across regions*, allowing shared expertise. Less strict rules and regulations around testing would enable flexibility in early phases of the development. Also, *training local experts to provide supervision*, assigning someone the role of *super-user (i.e.*,* staff members with advanced knowledge of the system who support colleagues in its use) and/or experienced deliverer that can offer support and advice*, was expected to strengthen the delivery of the digital intervention:

*You [should] have a responsible person in each region who provides training and supervision […]. The regions operate differently*,* so trying to coordinate training and supervision [on a national level] might be [difficult]. This may be something you have to test*,* to figure out how it could work. […] In the programs we have today*,* we usually provide training within the region; we have trained ‘experts’ in the regions who take responsibility for the training and supervision of younger colleagues.* (Participant G). Stakeholders also highlighted the significance of *legal agreements with companies for training*, and *clear formal agreement for approval process* of the digital interventions.

Next to these facilitators, several barriers were noted. *Fragmented decision-making and unclear approval processes* can often delay progress, as *various stakeholders can ‘veto’ a digital intervention* before implementation. *System change creates tension due to unfamiliarity*, particularly when transitioning from traditional face-to-face to digital care. In addition, *low awareness among staff of available digital solutions* and *unfamiliarity with delivering digital interventions* were known factors limiting adoption: *“It’s the healthcare staff who refer people to these solutions*,* and if you don’t know that they exist*,* well then you don’t refer to them”*. Another important barrier was the *lack of time for testing* of new digital interventions.

### Individuals

Several factors related to individuals’ knowledge, capabilities, and needs were seen as potentially influencing implementation. Stakeholders expressed a *need for research* to create an *evidence-based treatment* for chronic pain as a facilitator. To deliver or recommend the digital intervention, psychologists need *trust in the digital product*. Moreover, it was emphasized that *using digital treatment gets easier over time*, which could positively influence adherence in both patients and treatment deliverers. The *significance of deliverers’ beliefs in the intervention* emerged as a strong facilitator, since they are more likely to recommend it to their patients when they perceive the intervention effective: *“It is also how much you*,* as a practitioner*,* believe in [the digital intervention] if you are going to introduce it to the patient*,* because you have to feel that it is good and valuable”.*

As a barrier, *patients may not explicitly seek treatment for pain*, as they may not perceive pain as a condition that can be addressed through psychological or behavioral intervention. Instead, they may present with other concerns such as stress, anxiety: *“It is [the pain] such an integrated part of some [patients’] lives or personality that they do not see it as a problem anymore. It is more about stress or other problems*,* all the things that might be giving them the tension. But they do not come in saying*,* “How can you help me with my pain?” They come in saying*,* “I worry about my kid*,* I cannot sleep at night*,* I do not like my relationship.” They do not complain about their body or pain”.*

Additionally, patients might not be aware of the availability of this type of interventions, due to a *lack of signposting* (i.e., information about the availability of the intervention not reaching the potential recipient). *Concerns about patient engagement to use digital treatments*, particularly in relation to initiating treatment and maintaining active participation over time, were voiced. *Preferences for in-person treatment* were cited as a barrier, potentially reflecting uncertainty toward digital delivery of healthcare. Some stakeholders noted that *therapists’ own belief of in-person treatment being superior* to digital treatment could further increase resistance. Moreover, the *deliverer’s uncertainty about the suitability of digital interventions and how to inform patients about them* may interfere with implementation.

#### Implementation process

The *Implementation Process* domain captures constructs related to activities and strategies needed to implement the DAHLIA intervention. Definitions following the CFIR constructs are included below to enhance clarity.

*Teaming and assessing needs* (i.e., how team collaboration is organized and how the needs of stakeholders are identified and assessed prior to implementation): To support teaming, utilizing *existing collaborations* within and beyond regional healthcare setting was highlighted as a facilitator for teaming. Moreover, stakeholders emphasized the importance of *systematically assessing and addressing needs of both deliverers and recipients*, which in turn facilitates these needs assessments. In contrast, a barrier for needs assessment was the lack of systematic and proactive processes for identifying needs. Depending on the healthcare setting (specialized vs. primary care), the *lack of proper medical assessment and role clarity* (i.e., what is expected and needed from the intervention deliverer) prior to initiating the patient to a digital intervention was raised as a concern, with stakeholders noting that insufficient assessment of patients’ medical needs could raise concerns about the appropriateness of the intervention.

*Assessing Context* (i.e., collecting information to identify facilitators and barriers to the delivery and the utility of the intervention in a specific context): Stakeholders highlighted that successful context assessment requires *mapping and understanding local processes to tailor implementation* to each region’s workflows and structure: *“It is not possible to map one region and then think that the map applies [to all other regions]. It is the form that needs to be navigated based on the local terrain*,* the local process”.* It was emphasized that the *context assessment should be iterative*, allowing for ongoing refinement of the intervention as local conditions evolve.

*Planning:* This construct captures activities related to developing plans for how the intervention could be integrated into routine practice. Stakeholders mentioned the importance of *structured guidance for the deliverers to ensure fidelity* in providing the digital intervention. *Standardized and clear procedures and documentation* were viewed as essential to support delivery of the intervention. Stakeholders highlighted the value of *developing a dissemination strategy to navigate multiple actors* by *choosing delivery channels that maximize reach and usability*. Keeping the intervention ‘simple’ might enhance scalability.

*Tailoring Strategies:* Stakeholders emphasized the importance of *adapting implementation strategies to navigate administrative constraints while balancing user expectations*, ensuring that both deliverers’ and recipients’ needs were assessed during real-world implementation, *“We will definitely run into some friction between what the system can do and what the users would like us to do.”* (Participant G).

*Engaging:* This construct focuses on efforts to involve key stakeholders to support introduction and uptake of the intervention. Stakeholders emphasized the significance of *engaging with users in ongoing co-design to ensure person-centeredness* and *strengthen program design*, highlighting that involving the intervention deliverers and recipients in an early phase helps to align the intervention with real-world needs. *Implementers were considered the key source of learning about new digital interventions* available, enabling the dissemination of the information. Moreover, *practitioner involvement is essential for patient adherence*, particularly during the early phases of the intervention when guidance is particularly needed. *Introducing the type of delivery (i.e.*,* online/remote) of the treatment to the participants* was viewed as essential to prepare them for a digital format.

Several barriers to engagement were mentioned, including the *limited promotion of available digital interventions* and *inconsistent promotion directed at intervention recipients*, which might reduce awareness. Furthermore, the fact that *information about available new digital interventions between regions is limited*, resulting in uneven dissemination of the available programs, was seen as a hinderance. Other implementation barriers included *limited knowledge about existing digital solutions* among healthcare staff and *lack of awareness among patients*, which in turn might prevent potential uptake and engagement.

*Doing*: This construct refers to actions taken to test elements of the intervention. Stakeholders described an intended implementation approach characterized by early initiation and iterative real-world testing. *“This thing where you test a bit at a time so that you don’t run everything at once and then you have a thousand problems to fix. You do it gradually and involve the people who are going to use the program. (…) And then*,* you test maybe on smaller [scale]*,* one or a couple of units*,* or clinics*,* that get to test with real patients*,* when you feel that you are satisfied that it’s a good enough […] and then you pilot and evaluate it.”* (Participant G). Stakeholders emphasized that piloting and iteratively testing the intervention in real-world settings would allow identifying practical challenges early on and adjusting to improve usability. Rather than waiting for a fully finalized intervention, stakeholders emphasized the importance of refining the intervention through use in practice, preventing development of the intervention behind closed doors. As one participant stated: *“We actually dare to move forward*,* but without trying to make it perfect—aiming at [getting started instead of] working with it exactly as we’ve planned. […] Let real patients and our employees get to try it out as we go so it does not become the same old [thing]”* (Participant D).

*Reflecting, and evaluating:* This construct captures how stakeholders reflect on experiences, feedback, and observations. Stakeholders emphasized the importance of *regular feedback through quantitative information* to monitor implementation progress and support *continuous quality review and updates* to keep the intervention evidence-based and up-to-date. One barrier for evaluation in clinical research settings was the *difficulty in extracting data for large-scale evaluation*, since it is challenging to retrieve data especially on a broad scale, from the national 1177.se platform, due to GDPR (General Data Protection Regulation) regulations, data transfer agreements, and legal concerns.

## Discussion

This study aims to identify stakeholder reported barriers and facilitators for the implementation of a digital behavioral intervention for individuals with chronic pain in the Swedish healthcare system. The findings are interpreted within the CFIR framework, to provide an integrated understanding of anticipated implementation determinants. In total, 86 facilitators and 38 barriers were reported by the stakeholders. In general, successful implementation of the digital intervention appears to depend on interacting factors across multiple CFIR domains. At the intervention level, features such as flexibility and personalization were perceived as important facilitators. At the inner setting level, organizational readiness, awareness of available digital interventions, and clarity in approval and selection pathways were considered as critical determinants. At the outer setting level, national digital infrastructure (e.g., 1177.se) and interregional collaboration were identified as enabling structures.

Overall, findings suggest high readiness and openness to digital interventions. However, potential barriers were identified related to training/supervision, decision-making, and systematic needs assessment. While stakeholders expressed strong support for digital interventions and interregional collaboration, lack of staff awareness about available digital interventions, unclear or complex approval pathways regarding for selecting and implementing digital interventions, and limited dissemination processes represent challenges to integration into routine care without structured implementation efforts. These barriers suggest that successful implementation requires not only technical readiness but also has practical implications such as a need for clearer organizational structures (e.g., who informs who), active dissemination strategies (e.g., use of various communication channels), and targeted efforts to increase awareness among healthcare staff (e.g., trainings and information events).

The facilitators and barriers identified in this study are largely consistent with findings from previous implementation research on digital behavioral interventions for chronic pain [3, 4, 38]. At the intervention level, stakeholders emphasized the relevance of features to support patient engagement and accessibility, including greater level of personalization, a flexible design, an explicit focus on person-centeredness, and availability of multiple mode of communication (e.g., phone, video, messaging). This is in line with existing digital health literature, showing that tailoring treatment to meet individual needs results in higher acceptability and sustained usability [22, 40]. Also, stakeholders described a general interest and demand for digital intervention in Sweden, together with existing and potential interregional communication and collaborations to support implementation. These findings are also consistent with evidence showing that external policies, national digital infrastructure, and cross-organizational collaboration are key facilitators for successful implementation [3, 24].

The presence of a national digital healthcare platform (1177.se) provides a structured implementation context and setting, aligned with previous research showing that reduced technical burden facilitates the integration of digital interventions into routine workflows [25]. The use of trained local experts and “super-users” who provide supervision and support is consistent with studies demonstrating that local champions (i.e., individuals who actively promote and support the implementation of the digital intervention within their setting) and implementation leaders help maintain intervention fidelity [13]. Regarding the implementation process, findings align with existing research emphasizing the significance of agile and ongoing adaptation of digital interventions [27]. As such, patient and practitioner involvement is essential for patient adherence and prior studies show that human support enhances adherence and engagement [42]. Finally, stakeholders’ reports of limited promotion of available digital interventions reflect previous research showing that insufficient dissemination and lack of awareness among both intervention recipients and deliverers impact adoption [36].

### Implications for implementation strategies

Building on the implementation barriers and facilitators identified in this study and guided by the CFIR domains, several actionable implementation strategies can be proposed to support future integration of the digital behavioral intervention into the Swedish healthcare system. To support interpretation of these determinants, the Expert Recommendations for Implementing Change (ERIC) [26, 41] taxonomy was used conceptually to illustrate how identified barriers and facilitators could inform prospective implementation planning. Given the pre-implementation focus of the study, the strategies proposed are preliminary and context-specific, rather than formally selected using the matching tool. Table 2 presents illustrative examples of ERIC clusters and strategies that could inform future implementation planning of the DAHLIA intervention.

**Table 2:** Illustrative ERIC strategy clusters and strategies informed by CFIR-identified barriers and facilitators for prospective implementation of the DAHLIA intervention

CFIR Domain: Identified themes	Relevant ERIC strategy cluster	Illustrative ERIC strategies	Relevance for implementation of the DAHLIA intervention
*Intervention domain*: *Barrier*: Concerns about intervention recipient and deliverer preferences for face-to-face treatment	Develop stakeholder interrelationships	Identify and prepare champions	Specifically training new therapists aiming to strengthen confidence in the utility and effectiveness of digital interventions; where possible, have a face-to-face intake.
*Intervention domain*: *Facilitator*: The flexible, modular design of the digital intervention and the availability of multiple communication modes	Adapt and tailor to context	Promote adaptability	Allowing patients to engage with the treatment in their own speed, and by actively matching mode of contact to patient preferences.
*Intervention domain:* *Facilitator*: Balancing patient self-management with therapist support	Provide interactive assistance	Facilitation	Weekly patient-therapist contact to help balance patient autonomy with professional support and assistance where needed.
*Intervention domain:* *Barrier*: Limited language adaptability (potential barrier for migrant groups)	Adapt and tailor to context	Promote adaptability	Culturally and linguistically adaptating the treatment to increase reach (offering different versions online).
*Intervention domain*: *Facilitator*: Involving stakeholders early and engaging managers to demonstrate utility	Develop stakeholder interrelationships	Identify and prepare champions	Identify and involve ‘clinical coordinators’ per region, to ensure one point of contact when implementing nationally. Regularly ask for feedback from clinical coordinators.
*Inner setting:* *Barrier*: Low awareness among staff and unfamiliarity with delivering digital interventions	Train and educate stakeholders	Conduct ongoing training	Offering support to therapist/ clinical coordinators delivering the DAHLIA intervention upon request. Clinical coordinators and experienced therapists guiding more junior colleagues new to the DAHLIA intervention.
*Inner setting*: *Barrier*: Fragmented decision-making and unclear approval processes may impact implementation fidelity and timelines	Use evaluative and iterative strategies	Develop a formal implementation blueprint	Rigorously document and evaluate implementation processes in one region and utilize this knowledge to guide the implementation in another region.
*Inner setting:* *Facilitator*: Presence of trained local experts and super-users	Develop stakeholder interrelationships	Identify and prepare champions	Trained local experts and super-users (i.e., clinical coordinators) in every region that the digital intervention is implemented.
*Implementation process*: *Barrier*: Lack of knowledge and limited awareness of digital interventions among both intervention recipients and deliverers indicates the importance of systematic dissemination strategies	Engage consumers	Use mass media	Use Swedish newspapers, leaflets, digital newsletters, podcasts, or healthcare websites/ blog posts to share knowledge and updates about the intervention.
*Implementation process:* *Facilitator*: Strong readiness for iterative implementation and continuous adaptation	Use evaluative and iterative strategies	Stage implementation scale up	An agile, iterative approach to support ongoing refinement of the digital intervention.

Concerns regarding preferences for face-to-face treatment emerged as a potential barrier to engagement with digital intervention. This finding is consistent with previous research indicating that beliefs about intervention modality can influence uptake [3]. Educational efforts targeting intervention deliverers may therefore be particularly important, especially for those new to delivering digital interventions. In contrast, flexible and modular design of the digital intervention and the availability of multiple communication modes were seen as facilitators, aligning with evidence showing that flexibility in pacing and personalization can enhance user engagement [18]. These characteristics can be leveraged by identifying champions, promoting adaptability (e.g., allowing patients to progress at their own pace), and actively matching mode of contact to patient preferences.

Low awareness of available digital interventions and unfamiliarity with their delivery among staff were identified as barriers. These findings emphasize the relevance of training and supervision (e.g., offering support to therapist delivering the DAHLIA treatment upon request*)*, and providing clinical supervision (e.g., clinical coordinators and experienced therapists guiding more junior colleagues new to the DAHLIA treatment) [13]. In parallel, fragmented decision-making and unclear approval processes may impact implementation fidelity and timelines. These challenges highlight the need for developing a formal implementation blueprint [1]. The presence of trained local experts and super-users was identified as a key facilitator and can be strengthened through identifying and preparing champions.

Regarding the implementation process, lack of knowledge and limited awareness of digital interventions among both intervention recipients and deliverers indicates the importance of systematic dissemination strategies, including use of media. For example, newspapers, leaflets, digital newsletters, podcasts, or healthcare website blog posts might be useful, specifically when tailored to individuals with chronic pain. Stakeholders also emphasized strong readiness for iterative implementation and continuous adaptation, which aligns well with the agile implementation approaches. This approach can be facilitated by proceeding with implementation in stages and scaling up gradually [29].

### Integrating stakeholder feedback into the DAHLIA project

While the present study focused on identifying anticipated barriers and facilitators prior to implementation, insights from stakeholders have also informed refinement and optimization of the DAHLIA intervention in parallel agile development work [2]. In particular, feedback regarding the importance of flexible mode of communication (e.g., phone, video, messaging) and modular session design (e.g., micro-sessions) was incorporated into iterative development efforts, as described elsewhere [35]. Also, following each iterative testing, intervention recipients evaluated weekly modules upon completion, and exit interviews were conducted with both intervention deliverers and recipients. This feedback was used to refine treatment content, training materials, and further improve the iterations, and will be evaluated in a feasibility trial [2]. Furthermore, and in line with stakeholder input in the present study, local leaders in several pilot regions were engaged in the development and testing of the DAHLIA treatment, by providing support and supervision to intervention deliverers. Although this implementation work occurred outside the scope of the present study, and therefore not reported here as implementation outcomes, it illustrates how early stakeholder input may inform ongoing intervention refinement.

### Strengths and limitations

There are several strengths of the present study worth highlighting. First, the study identified barriers and facilitators early in the development and implementation process, allowing strategies to be designed proactively, which increases the likelihood of successful and sustainable adoption. Second, the comprehensive overview of anticipated barriers and facilitators of implementation integrated perspectives from multiple intervention agents, including healthcare professionals, managers, and IT developers. Third, the CFIR framework [8] provided a systematic and structured approach to understanding factors impacting implementation. Finally, relating the stakeholder input to the ERIC strategies allows for guidance on how to translate barriers and facilitators into practical implementation approaches.

Several limitations should be noted. The sample size was small (*n* = 8) due to the in depth and explorative nature of the study. The study deliberately focused on one region, as it is suggested to be specific when exploring the implementation context [30]. Thus, although the sample included multiple intervention agents and resulted in many implementation determinants, the generalizability of the findings is uncertain. Furthermore, the present study does not include input from intervention recipients. Instead, patients as end-users were address in another study within the DAHLIA project [35], where people with chronic pain participated in the focus groups to inform healthcare needs and design preferences for the digital behavioral intervention. Also, patient perspectives are explored in an ongoing feasibility trial. Ideally, the implementation success should be measured using implementation frameworks such as BIT model [17] and the RE-AIM framework [12], to assess key factors including reach, adoption, and maintenance.

### Conclusion

This study identified anticipated barriers and facilitators to the prospective implementation of a digital behavioral intervention for chronic pain by exploring the perspectives of stakeholders in a regional Swedish healthcare context. Guided by the CFIR framework, the findings highlight determinants across all CFIR domains, emphasizing the importance of flexibility in intervention design, ongoing training and support for deliverers, clear organizational structures, and proactive dissemination efforts.

Overall, this work contributes to the growing implementation science literature on digital health by demonstrating how early stakeholder perspectives can inform prospective implementation planning.

## Supplementary Information

Below is the link to the electronic supplementary material.

Supplementary Material 1

## Data Availability

The datasets generated or analyzed during the current study are not publicly available due to data privacy but are available from the corresponding author (AST) on reasonable request.

## Abbreviations

ACT: Acceptance and Commitment Therapy
CBT: Cognitive Behavioral Therapy
CFIR: Consolidated Framework for Implementation Research
DAHLIA: Digital behaviourAl HeaLth for chronIc pAin
ERIC: Expert Recommendations for Implementing Change
GDPR: General Data Protection Regulation
REDCap: Research Electronic Data Capture
