# Supplementary material for: Anticipated barriers and facilitators of implementing a digital behavioral treatment for chronic pain: a qualitative interview study
Source: BMC Health Serv Res. 2026 May 29;26:772. doi: 10.1186/s12913-026-14820-8 (PMC13222497; doi:10.1186/s12913-026-14820-8)
Supplement: Supplementary file 1 — Supplementary Material 1 [file 12913_2026_14820_MOESM1_ESM.docx]

**Multimedia Appendix 1:**

**Baseline interviews with stakeholders**

***Stakeholder: developers***

**I. General**

Theme: Experience and development of digital interventions within the 1177 web-platform

1. What is your job description and what are your responsibilities?
2. How is the 1177 web-platform structured, in the region of Kalmar and Sweden?
3. Who decides/has the overall responsibility for deciding which interventions should be developed and implemented?
4. How many digital interventions are available within 1177 in your region?
5. What kind of health areas do the digital interventions you know about focus on?
6. Who developed these interventions; who integrated them in the 1177 platform?
7. Who is responsible/ involved in the maintenance of the interventions?
8. If/ how is the interventions’ content updated?
9. How are these interventions financed?
10. If/ how are the interventions used and promoted in health care, and if so, how?
    - Who is responsible for deciding which interventions should be offered or possibly discontinued?
    - Who is involved in promoting the interventions?
11. What kind of feedback do you receive or collect during an intervention?
12. Are treatments evaluated overall, and if so, how it is done?
13. If/how do collaborations with other regions look like?

**II. Specifics (focus about DAHLIA project)**

We will now look at the implementation process of the DAHLIA intervention. By implementation, we mean the development of the intervention, its integration into the system, evaluation, and maintenance over time.

1. How would you describe the anticipated implementation process of this intervention?
2. What is needed to support the implementation process?
3. What could facilitate the implementation process?
4. What could hinder the implementation process?
5. What advantages/strengths do you see in the DAHLIA program?
6. Are you enthusiastic about this intervention, if so, why?
7. Do you think this intervention has the potential to be successful in your region, and Sweden?
8. Where would you like to see this intervention in 5 years?

***Stakeholder: health care managers***

**I. General**

Theme: Experience and promotion of digital interventions in care facility

1. What is your job description and what are your responsibilities?
2. How many digital interventions are currently offered on the 1177 platform in your region? Do you offer digital interventions via digital platforms other than 1177?
3. What is your involvement in digital interventions in your care facility? How do you support the use of digital interventions?
4. Who decides/has the overall responsibility for deciding which interventions should be developed and implemented?
5. What is needed to implement an intervention from the 1177 web-platform in your care facility?
6. How do digital interventions get financed in your care facility?
7. What hinders the implementation of these interventions, in your eyes?
8. If/ how does your care facility collaborate with other regions regarding digital interventions from the 1177 web-platform?

**II. Specifics (focus about DAHLIA project)**

1. Do you think there is a need for this intervention? Please elaborate.
2. What kind of benefits do you anticipate for employees through this intervention?
3. What kind of benefits do you anticipate for patients through this intervention?
4. If/ how are the interventions used and promoted in health care, and if so, how?
   1. Who is responsible for deciding which interventions should be offered or possibly discontinued?
   2. Who is involved in promoting the interventions?
5. How will you promote this intervention in your care facility?
6. Do you think this intervention has the potential to be successful in your care facility, and Sweden?
7. Where would you like to see this intervention in 5 years?

***Stakeholder: health care professionals***

**I. General**

Theme: Experience and use of digital interventions with patients

1. What is your job description and what are your responsibilities?
2. What is your experience in delivering interventions via the 1177 platform?
3. What makes it attractive to deliver such an intervention?
4. How are you usually informed that there is a new intervention available on 1177?
5. What resources are needed for you to deliver these interventions (e.g., time, knowledge, managerial support)?
6. What hinders you to deliver these interventions?

**II. Specifics (focus about DAHLIA project)**

1. Do you think there is a need for this intervention? Please elaborate.
2. What benefits for yourself/your work do you anticipate through this intervention?
3. What benefits for your patients do you anticipate?
4. What disadvantages or problems do you anticipate when delivering this intervention?
5. What would hinder you to deliver this intervention?
6. What would facilitate you to deliver this intervention?
7. Are you enthusiastic about this intervention, if so, why?
8. Do you think this intervention has the potential to be successful in your care facility?
